# Hemodynamics in Idealized Stented Coronary Arteries: Important Stent Design Considerations

**DOI:** 10.1007/s10439-015-1387-3

**Published:** 2015-07-16

**Authors:** Susann Beier, John Ormiston, Mark Webster, John Cater, Stuart Norris, Pau Medrano-Gracia, Alistair Young, Brett Cowan

**Affiliations:** Faculty of Medical and Health Sciences, University of Auckland, Private Bag 92019, Auckland, 1142 New Zealand; Mercy Angiography, 98 Mountain Rd, Mt Eden, Auckland, 1023 New Zealand; Green Lane Cardiovascular Service, Auckland City Hospital, Park Rd, Auckland, 1030 New Zealand; Faculty of Engineering, University of Auckland, Private Bag 92019, Auckland, 1142 New Zealand

**Keywords:** Hemodynamics, Coronary artery disease, Computational fluid dynamics (CFD), Stent design, Wall shear stress (WSS), Stent

## Abstract

Stent induced hemodynamic changes in the coronary arteries are associated with higher risk of adverse clinical outcome. The purpose of this study was to evaluate the impact of stent design on wall shear stress (WSS), time average WSS, and WSS gradient (WSSG), in idealized stent geometries using computational fluid dynamics. Strut spacing, thickness, luminal protrusion, and malapposition were systematically investigated and a comparison made between two commercially available stents (Omega and Biomatrix). Narrower strut spacing led to larger areas of adverse low WSS and high WSSG but these effects were mitigated when strut size was reduced, particularly for WSSG. Local hemodynamics worsened with luminal protrusion of the stent and with stent malapposition, adverse high WSS and WSSG were identified around peak flow and throughout the cardiac cycle respectively. For the Biomatrix stent, the adverse effect of thicker struts was mitigated by greater strut spacing, radial cell offset and flow-aligned struts. In conclusion, adverse hemodynamic effects of specific design features (such as strut size and narrow spacing) can be mitigated when combined with other hemodynamically beneficial design features but increased luminal protrusion can worsen the stent’s hemodynamic profile significantly.

## Introduction

Percutaneous coronary intervention (PCI) with stents is a widely used treatment for atheromatous coronary artery disease, a leading cause of death in the Western world.^42^ Yet, PCI failure is a relatively common occurrence, with 2% of stent patients dying due to thrombotic occlusion, and 15% requiring additional intervention for restenosis.^25^ Stent-induced hemodynamic changes are one of the important determinants of PCI outcome^18^ and stent design drives these hemodynamic changes.

A link between stent design and adverse clinical outcome was first established in stented rabbit iliac arteries,^9^ and this was followed by changes in WSS being associated with neointimal hyperplasia.^24^ Further investigations using simplified numerical^14^ and experimental methods,^2^,^7^ revealed that narrow strut spacing lead to undesirable flow stagnation zones and application of these concepts to 2D single stent units^8^ subsequently guided several computational studies. These found that strut spacing,^14^ stent connectors,^36^ strut peak angle,^12^ and strut thickness^15^ were all important hemodynamic considerations in stent design.

Strut thickness was the focus of many clinical studies with thicker struts causing higher thrombogenic risk,^20^ restenosis and reintervention rates.^18^ Subsequent computational studies however were more equivocal with some reporting thicker struts causing unfavorable flow regions,^15^,^23^ and others showing almost the opposite effect of reduced regions of adverse flow.^1^ Inter-strut spacing was found important and should be large to restore disturbed flow.^14^ Struts which are orientated to the flow direction appeared to reduce the area of flow recirculation. This includes connectors which were found only hemodynamically beneficial when aligned longitudinal to the flow.^14^ Computational optimization of the number of crowns revealed that the optimal number was dependent on the intra-strut angle^12^ (with 40° being ideal) and appeared to be independent of vessel diameter. This was subsequently refined by the same author, demonstrating that optimal strut angle may be somewhat dependent on vessel size.^11^ Peak-peak or valley-peak alignment also appeared to influence the ideal angle. These studies were often simplified using 2D,^16^ geometric simplifications,^8^,^14^ a smaller number of stent cells,^15^ and/or steady-state^23^ models.

Commercially realistic stent designs have also been investigated^1^,^10^,^11^,^32^,^36^ focusing primarily on the comparison of hemodynamic parameters^11^ and ranges^32^ rather than linking design features to hemodynamic observations. These studies have several limitations: (1) multiple design features were varied at once, limiting the understanding of each design feature’s contribution to the altered flow,^11^ (2) the focus was on a single design feature only,^36^ (3) observations changed throughout the cycle leading to inconclusive results,^1^ and (4) similar stent designs were used when design parameters were considered in greater detail.^10^

Stent apposition is clinically desired during stent deployment but imposes adverse mechanical stimuli on the vessel wall.^19^ The present study hypothesizes that stent protrusion rates into the lumen also alter the hemodynamic profile—an aspect of stent performance which has not previously been considered. Full stent malapposition, that is under-deployment with stent struts fully exposed to the blood flow, has shown to increase thrombogenicity.^43^ Only the hemodynamic impact of idealized single strut malapposition was studied before.^37^

A variety of *in*-*vivo* features impact on the computational prediction of hemodynamic flow including: vessel curvature,^22^ compressive force of the stent,^3^ local deformation, and associated tissue prolapse.^27^ These considerations are vessel and lesion specific, and vary with the degree of disease, presence of tissue calcification and vessel geometry. Along with a limited ability to quantify the anisotropic vessel wall properties, this leads to significant assumptions being made in simulations. The goal of the present study was to determine the hemodynamic impact of major stent design parameters to inform stent development. We therefore focused on idealized stent geometries to elucidate the underlying aspects of design by eliminating local deformations occurring as a result of stent deployment, vessel curvature, and tissue prolapse.

The novelty of the present study therefore resides in the investigation of the hemodynamic impact of specific stent design features including strut spacing, stent size and luminal protrusion rates to full malapposition, with findings being applied to two commercially available stent designs.

## Methods

### Idealized Coronary Artery and Simplified Stent Geometries

An average geometry of the proximal left main artery was derived from 101 asymptomatic angiography cases (average age 54 ± 8 years; 57 females),^29^ which yielded an average diameter of approximately 4 mm. Two commonly available commercial stents, “Omega” (Boston Scientific, Marlborough, MA, USA), and “Biomatrix Flex” (Biosensors International, Singapore), both 10 mm in length, were deployed *ex*-*vivo* in a straight silicone vessel of 4 mm diameter. These were then scanned using micro-computed tomography with Skyscan-1172 (Bruker Biosciences Corporation, Billerica, MA, USA) with an isotropic voxel resolution of 0.3 µm to obtain detailed geometric information, from which an idealized stent geometry was derived. The computer aided design software Autodesk Inventor Professional 3D (Autodesk, San Rafael, CA, USA) was used to correct local stent deformations and smooth the geometry. This procedure resulted in an “as manufactured” stent geometry with a deployed diameter matching the test vessel, rather than a deployed geometry with local deformations. Variations in strut cross-sectional shape causes changes in hemodynamic behavior,^16^ so all cross-sections were simplified to have a circular shape for the purpose of this study. Vessel walls were circular with constant diameter and smooth luminal surfaces. The resulting flow domain was derived and imported into ANSYS Workbench 14.5 (ANSYS Inc., Canonsburg, PA, USA).

### Computational Fluid Dynamics (CFD)

ANSYS Meshing 14.5 was used to create a patch-conforming, unstructured tetrahedral mesh with variable mesh spacing to represent the small and rapidly changing features in the stent region. The mesh size was chosen to accurately model the shearing at the wall and was optimized (<4% relative error for 100% greater element density) to comprise approximately 3 million elements for the strut spacing analysis and approximately 8 million elements for the more complex analyses of strut size, luminal protrusion, and specific stent designs. ANSYS CFX 14.5 was used to solve the CFD simulation, using a high performance parallel computing cluster (New Zealand eScience Infrastructure, 64-bit 2.7 GHz Intel Xeon, 60CPU, 40 GB RAM) and computation times are provided in Table 1. Although a quarter or half-section domain analysis would have been sufficient in some cases, we performed a full three-dimensional analysis for future direct comparison with experimental studies.

**Table 1:** Computation time for each simulation.

Case study	Type	Computation time (h)
Strut spacing	All	<3
Strut size	81 µm	27
	120 µm	16
Protrusion	25%	35
	50%	25
	75%	14
	Malapposed	5
Stent comparison	Omega	27
	Biomatrix	25

As accurate stent geometry modeling is computationally extensive, the high performance computing facility ‘NeSI’ was utilized. This is a shared facility of a number of supercomputing clusters, and thus overheads and competing demand can vary significantly. For this reason no overheads are reported. Computation times are reported for a four node cluster with 40 CPU and 20 GB memory each. Thus, the total number of core hours can be estimated by multiplying the reported times by 160

**Figure 1:**
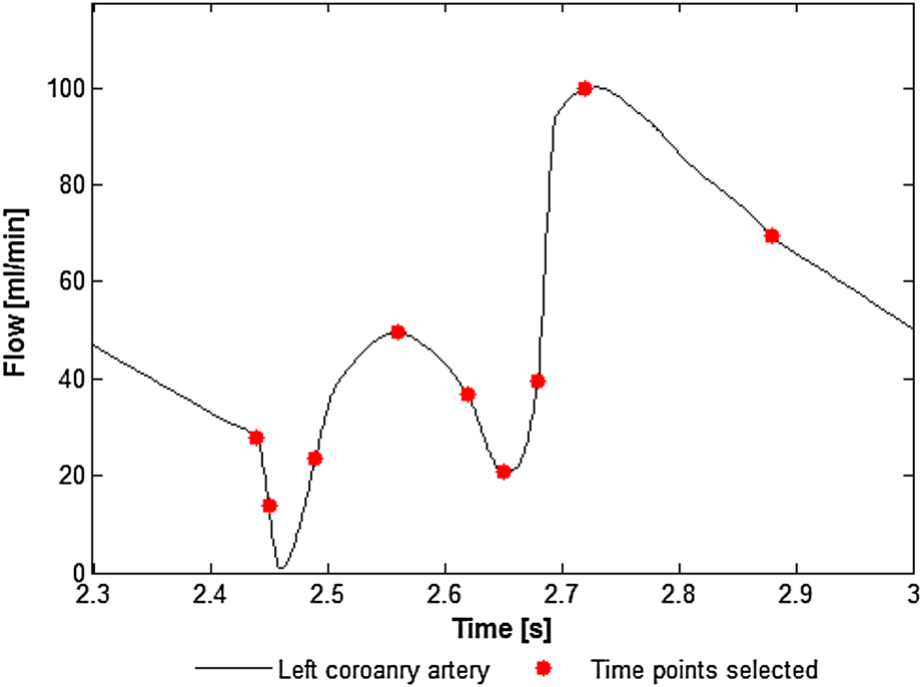
Left main coronary blood flow over time prescribed as the inlet boundary condition (adapted from Nichols *et al*.^33^). Red circles indicate timepoints selected for transient analysis throughout the manuscript taken from the fourth simulated cardiac cycle.

The shear thinning behavior of blood was accounted for using the non-Newtonian “Carreau-Yasuda” model as recommended in the literature.^30^ Blood density was assumed to be 1050 kg/m.^3^ An inlet boundary condition of flow rate vs. time (ranging from 0–102 mL/min) was adapted from Nichols *et al*.^33^, assuming a heart rate of 75 beats/min (Fig. 1). The bulk Reynolds number (*Re*) was approximately 80. Due to the simplification of using straight vessel geometries, a parabolic, laminar flow profile was used at the inlet boundary and the entrance length extended by 36 mm (0.06*Re* × 4 mm vessel diameter) to ensure fully developed flow. A constant static pressure condition was prescribed at the outlet.

Transient simulations consisted of four consecutive cardiac cycles. Results were derived only from the fourth cycle to minimize transient start-up effects. A time step of 0.001 s was used to ensure a Courant number (*Cr*) below 5, with every 5th time step saved for subsequent analysis. A laminar numerical fluid model governed the simulation solution with residual targets of 10^−4^ (a stricter residual target of 10^−6^ resulted in a 0.02% change in RMS flow and increased computation time from 10 to 26 h computation time) and a maximum of 5 coefficient loops as convergence control. No-slip boundary conditions were applied on all internal surfaces. The geometries were rigid as stent deployment and calcification of the arterial wall stiffen the vessel^28^ and a recent study showed little difference between rigid-wall and compliant fluid-structure simulations.^4^

### Simulations

**Figure 2:**
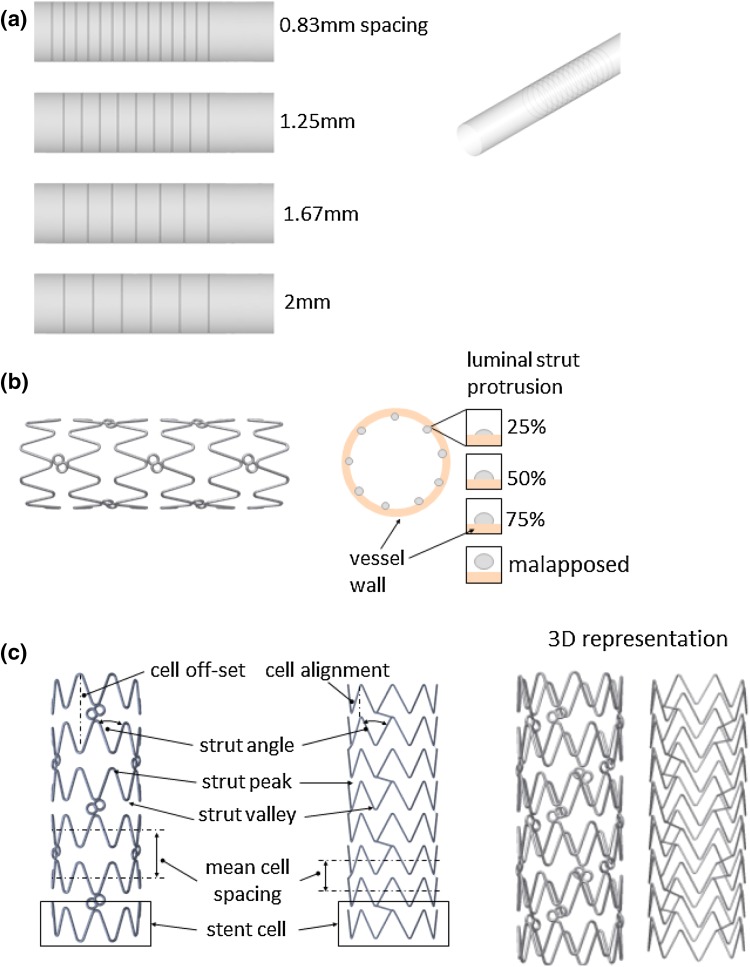
(a) Struts with 0.83, 1.25, 1.67, and 2 mm spacing (upper to lower) modeled with 81 and 120 µm strut sizes. (b) Biomatrix geometry with 25, 50, and 75% luminal protrusion and malapposition of the stent protruding into the vessel lumen. (c) Biomatrix (left) and Omega (right) geometries with stent design labels.
II. Strut size: The Omega stent geometry was modeled with a manufactured strut size of 81 µm and a hypothetical thicker strut size of 120 µm for comparison.
III. Stent protrusion: Luminal protrusion of the Biomatrix stent was studied (Fig. 2b) with the stent apposed with 25, 50, and 75% of the stent cross-section in the flow domain, and the remainder embedded in the vessel wall (Fig. 2b). In a second phase, a completely malapposed Biomatrix stent was simulated with a gap of 0.29 mm between the stent edge and vessel surface was simulated.
IV. Stent comparison: The Biomatrix and Omega stent geometries were modeled with a 50% luminal protrusion which resulted in the Omega stent’s connectors being fully embedded in the vessel wall. The Omega geometry has 81 µm struts, 1.4 mm mean cell spacing, diagonally aligned straight connectors embedded in the vessel wall, and 8 strut peaks per cell which were aligned from cell to cell. The Biomatrix geometry has 120 µm struts, 1.6 mm mean cell spacing, 9 strut peaks per cell with a radial peak offset from cell to cell, and three approximately circular connectors per cell set. Both stents were 10 mm long and 4 mm in diameter (Fig. 2c).

The following simulation studies were conducted:

I. Strut spacing: Eight straight vessels with simplified stent geometries (regularly spaced circumferential rings with spacings of 0.83, 1.25, 1.67, and 2 mm) were created with strut sizes of 81 and 120 µm. The stent length was 10 mm, with a 4 mm diameter and no connectors were modeled (Fig. 2a).

### Hemodynamic Metrics

In order to understand the effects of hemodynamic changes, the three most established hemodynamic metrics were studied: wall shear stress (WSS), time averaged WSS (TAWSS), and WSS gradient (WSSG). Low WSS and TAWSS were studied due to their strong relevance to atherogenesis, and high WSS and WSSG due to their recent emergence as key regulators of vascular pathophysiology.^6^

#### Wall Shear Stress (WSS)

Common atheromatous disease locations characteristically have low WSS. Intimal hyperplasia is promoted by the release of tissue growth factors at WSS < 1.5 Pa.^26^ Atherosclerotic intimal thickening increases with lower WSS and regions with WSS < 0.5 Pa are prone to atherosclerosis, although this is typically a stiffer and more stable plaque phenotype.^26^ Areas of coronary arteries with WSS greater than 1.2 Pa have been found to have less atheromatous narrowing^40^ and more positive remodeling.^39^ In contrast, an intravascular ultrasound study found an increase in the necrotic core area for WSS > 2.5 Pa,^38^ suggesting that the development of a more vulnerable disease phenotype with plaques more prone to rupture, and consequent thrombus and occlusion. It is possible that sites of low WSS are prone to atheromatous lesion formation, whereas sites of high WSS may be at increased risk of plaque rupture and thrombosis.^6^

Ku^21^ established that adverse vascular shear environments represent a continuum and demonstrated unfavorable behavior from <1 Pa (whereas 0.5 Pa had commonly been accepted as the cut-off). The same continuum is likely to exist for adverse high WSS behavior. There may be an intermediate “ideal” WSS range of approximately 1–2 Pa.

#### Time Averaged Wall Shear Stress (TAWSS)

TAWSS is WSS averaged over the cardiac cycle. Low TAWSS is associated with endothelialization of stent struts, with levels <0.5 Pa associated with cellular proliferation, intimal thickening, and inflammation.^21^,^24^,^41^

#### Wall Shear Stress Gradient (WSSG)

Rapid changes of WSS over short distances are quantified by the WSSG. Regions of high WSSG (>200 Pa/m) have been linked to intimal hyperplasia, formation of atherosclerotic lesions, and increased vessel wall permeability; and accelerate platelet activation and thrombus formation.^5^

The following thresholds were therefore considered to be unfavorable for the purposes of this study: (i) WSS < 0.5 Pa, (ii) WSS > 2.5 Pa, (iii) TAWSS < 0.5 Pa, and (iv) WSSG > 200 Pa/m. The present study uses CFD to determine WSS, TAWSS, and WSSG changes caused by variations in stent design parameters such as strut spacing, strut size, stent luminal protrusion, and specific stent geometry.

## Results

**Figure 3:**
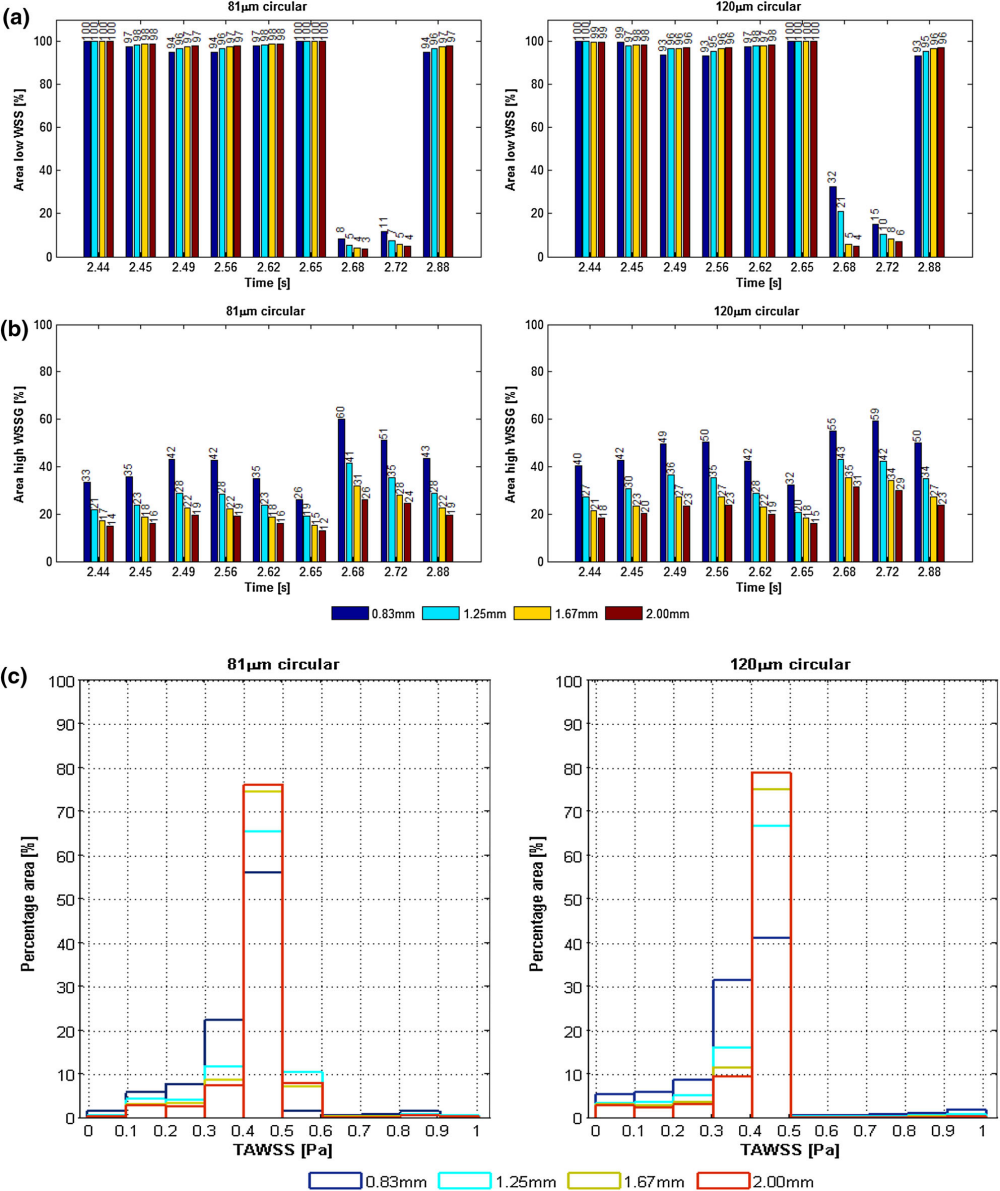
Strut spacing: (a) Percentage area of adverse stress over the cardiac cycle; WSS < 0.5 Pa (top) and WSSG > 200 Pa/m (bottom), for 81 (left) and 120 µm (right) strut sizes for all strut spacings. (b) TAWSS histogram for 0.83 mm (blue), 1.25 mm (cyan), 1.67 mm (yellow), and 2 mm (red) strut spacing with 81 µm (left) and 120 µm (right) strut sizes.

**Figure 4:**
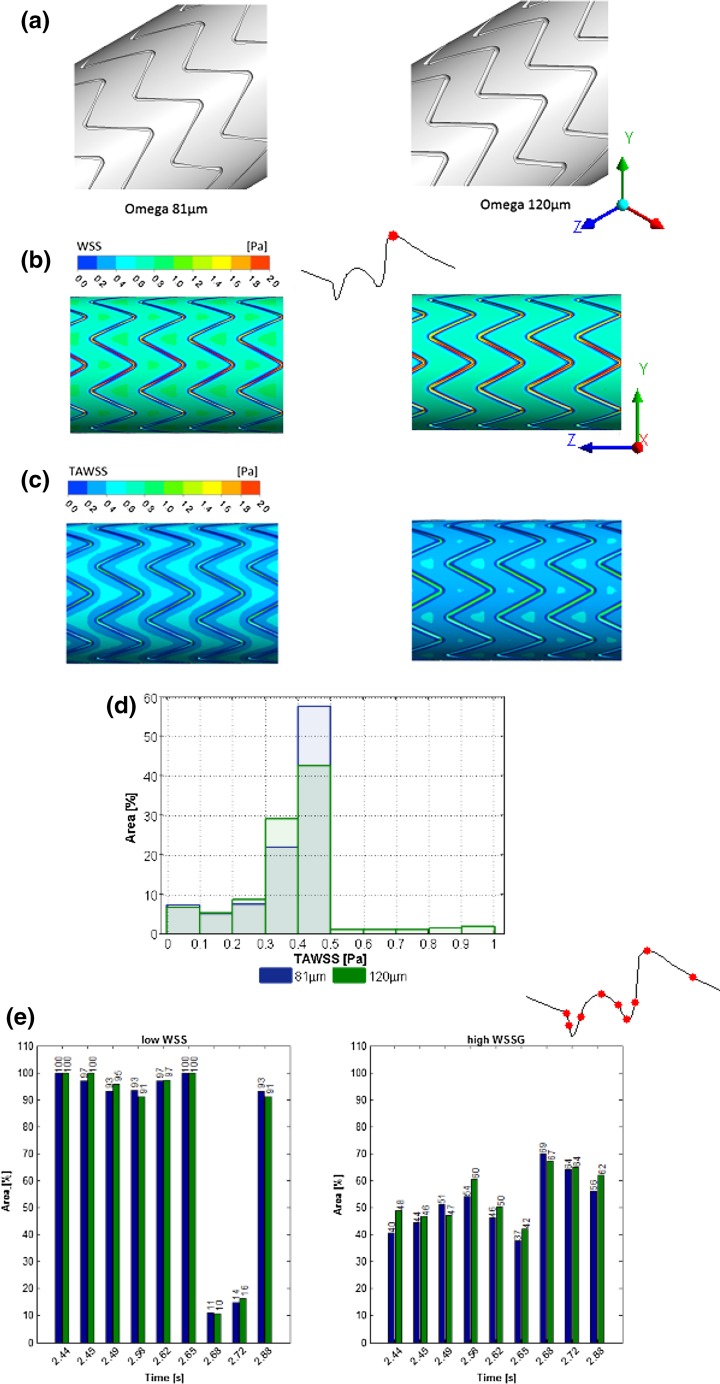
Strut size: (a) Omega geometry with 81 µm (left) and 120 µm (right) strut size, (b) WSS, (c) TAWSS contour for 81 µm (left) and 120 µm (right) strut size, (d) histogram of TAWSS distribution and (e) percentage areas of low WSS (<0.5 Pa, left) and high WSSG (>200 Pa/m, right) over the cardiac cycle for the 81 µm (blue) and 120 µm (green) Omega geometry.

**Figure 5:**
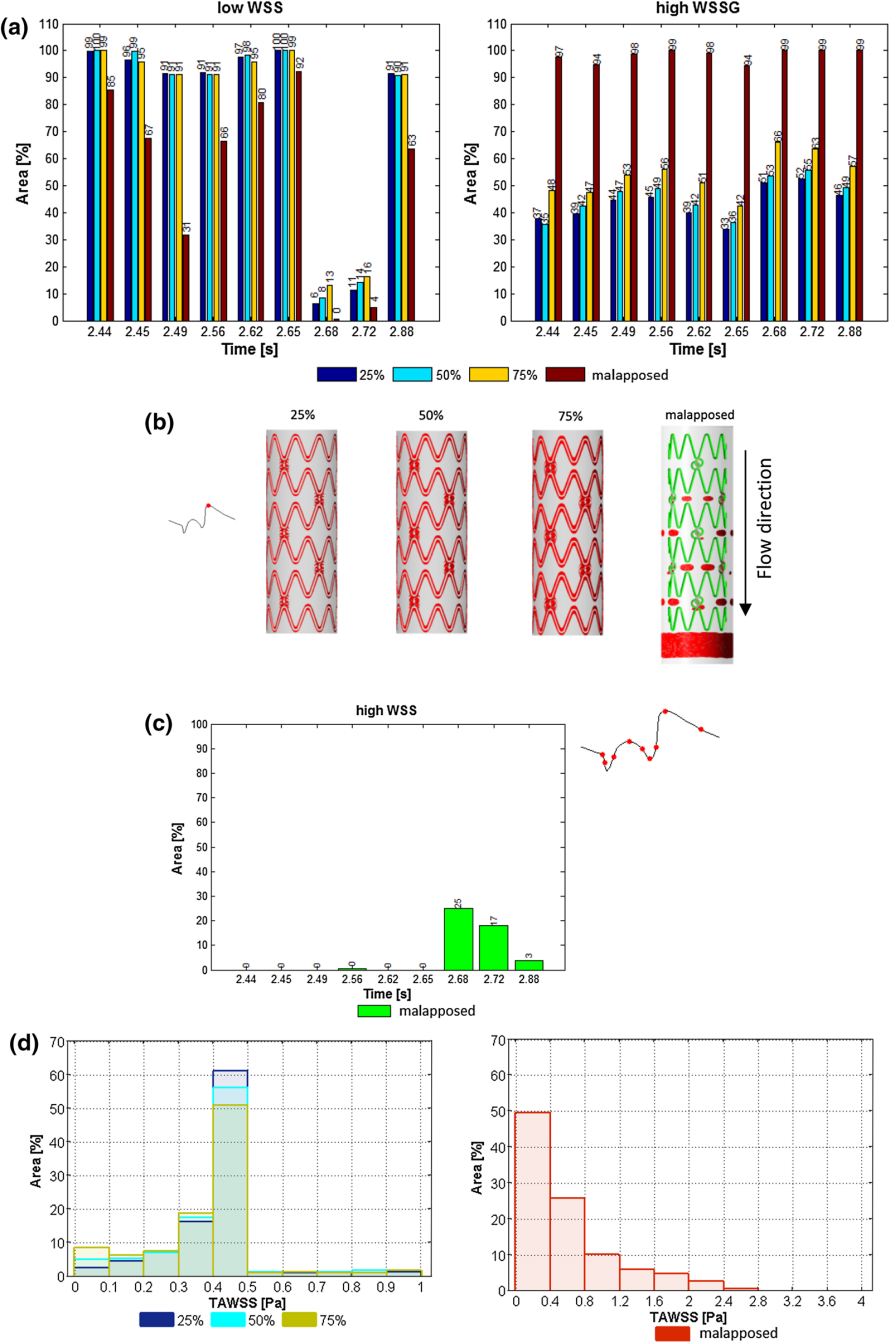
Stent protrusion comparison between 25, 50, 75% luminal protrusion and malapposition: (a) Percentage area of adverse haemodynamic parameters (WSS < 0.5 Pa and WSSG > 200 Pa/m) over cardiac cycle. (b) Areas of WSS < 0.5 Pa (red) and >2.5 Pa (green) for 25–75% luminal protrusion and malapposition (left to right) at peak flow (2.72 s). (c) High WSS > 2.5 Pa for malapposition over the cardiac cycle. (d) TAWSS distribution for 25, 50, and 75% luminal protrusion (left) and malapposition (right).

**Figure 6:**
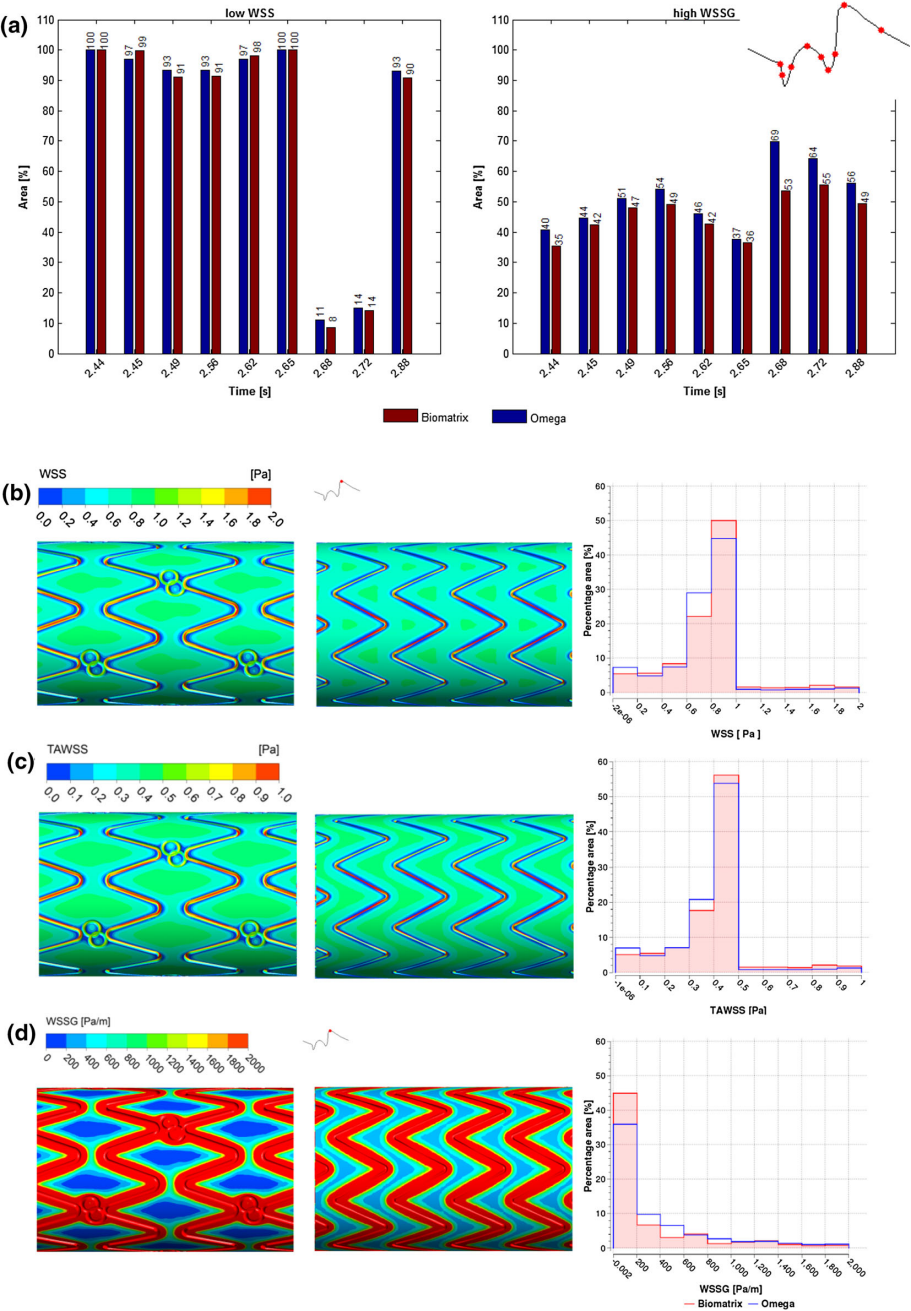
Biomatrix vs. Omega: (a) Area of adverse low WSS (<0.5 Pa, left) and high WSSG (>200 Pa/m, right) for Biomatrix (red) and Omega (blue) over the cardiac cycle. (b–d) Comparison between the Biomatrix (left panel) and Omega (center panel) geometries with histograms (right panel, where the Biomatrix is shown in red, and Omega in blue) of (b) WSS at peak flow (2.72 s); (c) TAWSS; and (d) WSSG at peak flow (2.72 s).

Table 2 summarizes the area-averaged statistical quantities of the TAWSS distribution and the percentage area of TAWSS < 0.5 Pa. Figures 3a, 4e, 5a, and 6a show the percentage area meeting the adverse hemodynamic criteria of low WSS (<0.5 Pa), and high WSSG (>200 Pa/m) at different times points in the cardiac cycle. Endothelial cells respond to shear stress^26^ and ideally cover the stent surface area within a few days after PCI.^16^ For this reason, the area considered is vessel and stent surface plus 5 mm of vessel on each side of the stent to capture any proximal or distal flow disturbances.

**Table 2:** TAWSS distribution and area-averaged statistical quantities.

	Strut spacing (mm)	Stent type	Strut size (µm)	Luminal protrusion (%)	TAWSS (Pa)				
					Mean	SD	Skewness	Kurtosis	Area < 0.5 Pa (%)
Strut spacing—81 µm strut size	0.83	R	81	50	0.30	0.11	0.46	8.07	95.0
	1.25	R	81	50	0.42	0.12	0.29	11.76	96.7
	1.67	R	81	50	0.42	0.11	0.16	14.69	97.4
	2	R	81	50	0.43	0.10	0.05	16.78	97.8
Strut spacing—120 µm strut size	0.83	R	120	50	0.38	0.16	0.46	5.66	93.3
	1.25	R	120	50	0.40	0.13	0.28	8.28	95.6
	1.67	R	120	50	0.41	0.13	0.79	12.49	96.6
	2	R	120	50	0.42	0.12	0.74	14.24	97.0
Strut size	1.44	O	81	50	0.39	0.17	0.75	7.15	93.5
	1.44	O	120	50	0.39	0.17	0.74	6.00	91.4
Luminal protrusion	1.60	B	120	25	0.41	0.15	0.56	6.57	92.0
	1.60	B	120	50	0.39	0.16	0.29	5.49	92.0
	1.60	B	120	75	0.38	0.18	0.68	11.36	91.6
	1.60	B	120	100	0.64	0.51	1.79	5.57	65.5
Stent comparison	1.44	O	81	50	0.39	0.17	0.75	7.15	93.5
	1.60	B	120	50	0.39	0.16	0.29	5.49	92.5

R, ring shaped; B, biomatrix; O, Omega

### Strut Spacing

Figure 3a shows the percentage area of adverse stress vs. strut spacing for 81 and 120 µm thick struts.

Narrow strut spacing was the main cause of adverse WSSG over the whole cycle and its area approximately doubled when the strut spacing was reduced from 2 to 0.83 mm. Similar effects were found for adverse low WSS around peak flow (2.68 and 2.72 s). Strut thickness had a smaller impact on both adverse WSSG and WSS regions. Adverse low WSS coverage was between 94 and 100% during the rest of the cardiac cycle. It is hence not surprising that TAWSS area below <0.5 Pa (Table 2) was similar for all spacings, demonstrating that momentary WSS effects are usually small when averaged over the cardiac cycle. Larger differences were found for the detailed TAWSS distributions with lower TAWSS for narrower spacings, especially for thicker struts (Fig. 3b).

### Strut Size

Thinner struts (Fig. 4a) represent smaller obstacles to the flow and allow for more rapid flow recovery.^16^ Higher near-wall velocities are therefore generated between cells (with slower central lumen velocities that are consistent with conservation of flow), with a consequent increase in WSS. Figure 4b shows the difference in WSS between both strut sizes at peak flow (2.72 s), demonstrating that the strut size affects the WSS distribution between strut cells with thinner struts leading to higher WSS between cells. The effect is even more apparent for TAWSS (Fig. 4c). Figure 4d shows that the thicker struts shifted the distribution to lower TAWSS values. The threshold of TAWSS < 0.5 Pa is close to the statistical mode of the distribution (kurtosis 7.1 for 120 µm vs. 5.9 for 81 µm) so the area results are sensitive to small changes in geometry (and to the cut-off chosen). The threshold analysis therefore did not reflect the unfavorable distribution effect (91.4% for thicker vs. 93.5% for thinner struts, Table 2).

### Stent Protrusion

Greater luminal protrusion generally caused larger areas of adverse WSSG throughout the cardiac cycle for all apposed cases (30–60% area for 25, 50, and 75% protrusion respectively) and this increased significantly when the stent was fully malapposed (undersized) with nearly 100% area (with area normalized to account for stent and vessel surfaces) for all time points analyzed (Fig. 5a). Apposed protrusion (25, 50, and 75%) also generated areas of adverse WSS < 0.5 Pa immediately adjacent to the struts at peak flow (red areas in Fig. 5b at 2.72 s). There were no areas of adversely high WSS (>2.5 Pa) for apposed stents. However, once the struts were fully protruded, the entire stent surface exhibited shear stresses >2.5 Pa (shown in green in Fig. 5b) around peak flow at 2.68, 2.72 and 2.88 s (Fig. 5c). In this case, the adverse low WSS regions (<0.5 Pa) encompassed the entire vessel after the stent for approximately one longitudinal cell length (Fig. 5b, right, shown in red). Conversely, the adverse low WSS at the vessel wall was reduced to small circular regions (red) located perpendicular to the regions between strut peaks, beginning at the second cell and growing in area in the flow direction (Fig. 5b). The malapposed stent had higher TAWSS (Fig. 5d, right) as expected from the WSS data.

For apposed stents, minor changes were found for adverse TAWSS areas (Table 2) but the TAWSS distribution shifted to lower TAWSS values with increased luminal protrusion. Again the statistical modes were close to the threshold of <0.5 Pa (Fig. 5d).

### Stent Design Comparison

**Figure 7:**
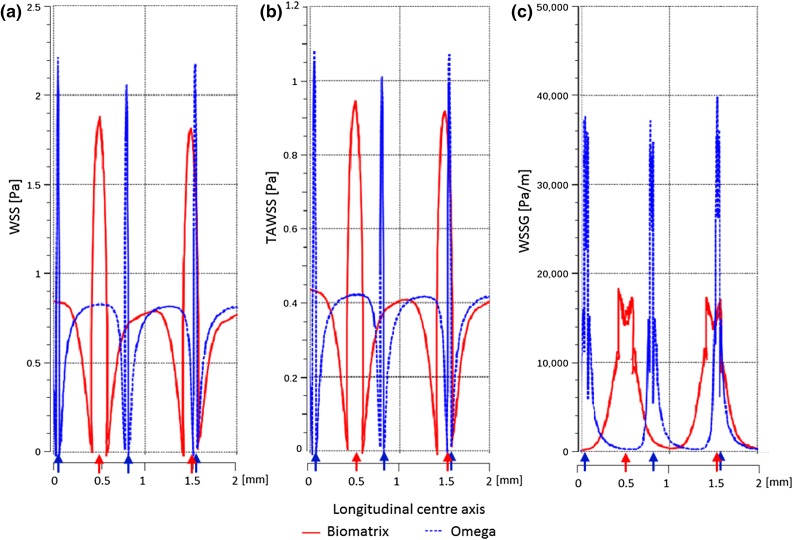
WSS at peak flow (2.72 s, left), TAWSS (middle), and WSSG at peak flow (2.72 s, right) along the longitudinal axis of the vessel, crossing the strut peaks for the Biomatrix (red) and Omega (blue) geometries for the first struts to 2 mm within the stented region. Strut positions are indicated by arrows.

Flow induced stress differed between the two simplified clinical stent geometries and showed less favorable values for the Omega geometry compared to the Biomatrix (Figs. 6a and 7). Omega showed consistently greater areas of adverse WSSG > 200 Pa/m over the cardiac cycle with up to a 16% increase in area when flow accelerated (2.68 s) to peak flow. High WSSG values were located immediately adjacent to the stent struts (Fig. 6d). Areas of low WSS < 0.5 Pa were slightly larger for Omega during most of the cycle (but not when flow velocity rapidly reduced at 2.45 and 2.62 s).

Figure 7 shows WSS, TAWSS, and WSSG along the longitudinal axes of the Biomatrix and Omega geometry with strut positions indicated by arrows on the *x*-axis. Peaks in WSS and TAWSS were slightly higher for Omega and occurred at the struts, while WSSG peaks were significantly higher for Omega (17,000 for Biomatrix vs. 39,000 Pa/m for Omega) and were located immediately proximal and distal to the struts. The Omega’s higher WSSG peaks indicate that the WSS changed more rapidly over short distances. Struts represent obstacles to blood flow, which stagnates upstream of the strut causing reduced WSS and TAWSS. Recirculation zones are created with changing flow direction proximal and distal to the strut resulting in high WSSG values. The flow at the exposed crest of the strut is higher than at the base, creating peak WSS and TAWSS stress. Between struts, the flow and shear quantities nearly recovered for both stents.

## Discussion

### Design Parameters

#### Strut Spacing

Narrower strut spacing has previously been found to be hemodynamically adverse.^14^ Similarly, the present study showed narrower strut spacing created areas of unfavorable low WSS and high WSSG. It was also demonstrated however, that thicker struts have an additional secondary effect but this can be mitigated by widening the strut spacing. Both thicker struts and narrower spacing led to low near-wall velocities (and higher velocities in the central flow), with a consequent reduction in WSS. Previous studies are contradictory about the importance of strut size,^1^,^15^,^17^,^20^,^23^ and the present findings may explain some of these differences. Our results suggest that a critical strut-size to strut-spacing relationship exists and previous studies show this may be linked to vessel size.^14^,^18^,^25^ Here, in the case of a 4 mm diameter stented vessel, similar stress values were found for a strut spacing of 1.67 mm with 120 µm strut, and 1.25 mm with 81 µm strut (Fig. 3a).

Adverse TAWSS area <0.5 Pa was universally high. However, the TAWSS distributions indicated that narrow stent spacing shifts the TAWSS distribution toward lower values (<0.4 Pa), especially for thicker struts. This may indicate another link between strut spacing and strut size where, for greater strut spacing (here ≥1.25 mm), strut size becomes less important for TAWSS.

#### Strut Size

Clinical observations on the significance of strut thickness are equivocal,^17^,^20^ as are computational studies on hemodynamic significance: A reduction in adverse WSS areas (87%) was found for 56 vs. 96 µm,^23^ while an increase in adverse WSS areas was reported for 50 vs. 150 µm stents.^1^ In our study, low WSS area for the manufactured Omega 81 µm and a hypothetical thicker strut of 120 µm were similar (15.4 vs. 16.3% area). These differences may be due to the use of only Newtonian fluid properties and steady state solutions,^23^ single cell research with deployment deformation^1^ or strut sizes differences. Even though the differences for all hemodynamic stress thresholds were found to be small, a tendency was demonstrated for thicker struts to reduce TAWSS between stent cells and shift the TAWSS distribution (Figs. 4c and 4d). This is not reflected in the TAWSS threshold comparison (Table 2), suggesting that the simple threshold of <0.5 Pa is dependent on the specific conditions of the study and may not always deliver an accurate indication if flow is favorable or unfavorable. Similar observations have been made in the literature.^32^

#### Stent Protrusion

Even when struts are apposed, adverse WSS and WSSG increased with luminal protrusion. This may indicate that greater luminal protrusion creates unfavorable flow, whereas a well-embedded stent (25% luminal protrusion) has less adverse hemodynamic effect. This is also demonstrated in the TAWSS distribution, which shifted to lower, unfavorable values for increased protrusion rates (Fig. 5d). In conjunction with the previous presented findings it can be hypothesized that strut thickness and spacing may have a stronger effect for increased luminal stent protrusion, which represents an area of future study.

#### Stent Design Comparison

The simplified Biomatrix geometry generally showed a more favorable hemodynamic stress profile than the Omega design.

Considering the results of the strut size experiment, the greater strut size of the Biomatrix stent would be expected to generate larger regions of adverse WSSG and lower TAWSS values. However, the WSSG observed was actually higher for the Omega stent and TAWSS distributions were similar. This suggests that either other design features outweigh the hemodynamic impact of strut size, and/or the strut spacing was large enough to mitigate the effect of the thick struts (see “Strut Spacing” section). The difference in mean cell spacing is small (1.4 mm for Omega and 1.6 mm for Biomatrix). These values can be misleading however, as the Biomatrix cells are offset which creates larger gaps (diamond shaped) between cell peaks rather than a consistent distance for the aligned Omega cells. Figure 6d demonstrates this by showing WSSG at peak flow (2.72 s), where the large inter-cell Biomatrix gaps lead to reduced spatial gradient of WSS and allow the generation of favorable WSSG < 200 Pa/m between cells. For Omega however, the inter-cell distance is uniform and not great enough for recovery to WSSG < 200 Pa/m. Similarly, Fig. 6b shows larger areas of higher WSS between cells for Biomatrix compared to Omega at peak flow (2.72 s). Thus, the Biomatrix stent’s larger strut thickness may be mitigated by the larger strut distance created by the cell offset.

Other design features also contribute to the hemodynamic profiles, such as the number of peaks and connectors in the stent. Biomatrix has nine strut peaks per cell which leads to geometrically narrower peaks (smaller angles) that are relatively more “flow-aligned” compared to Omega which has eight strut peaks. Previous research has demonstrated that for aligned cell designs like Omega’s, fewer peaks can adversely affect TAWSS due to greater flow misalignment (struts are more cross-flow directed) and this was found to outweigh the competing factor of minimizing stent-vessel area.^11^ For Biomatrix with the offset (peak-to-valley) design, more cell peaks increase the stent-to-vessel area, but also result in a better flow alignment of the struts.^11^ Thus, it is hypothesized that the greater hemodynamic stresses at the Omega struts (Fig. 7) were also caused by its less flow-aligned design of eight peaks with wider strut angles (56°) compared with the Biomatrix stent’s nine peaks with narrower strut angles (32°).^12^,^14^ This could explain why TAWSS is similar rather than less favorable as would be expected from the effects of its thicker struts.

The hemodynamic profiles did not change throughout the stented vessel region. This suggests that higher restenosis risk in the proximal stented vessel segments^19^ might be stent design independent, but this needs to be investigated further.

Theoretical stent design testing^13^ showed a similar TAWSS difference (16% greater area of TAWSS < 0.4 Pa compared with 11% in this study) for a stent with peak-peak alignment (as in the Omega design) relative to the valley-peak alignment (like Biomatrix’s design). It is important to note however that the stents studied differed in many other attributes (primarily open vs. closed, while here both Omega and Biomatrix are open designs).

The Omega stent’s connectors were considered to be embedded in the vessel wall, meaning its strut-vessel ratio was not fully represented and this may have led to a minor overestimation of its performance.^14^,^36^ It is also likely that Omega’s strut alignment with reduced inter-strut spacing outweighed the advantages of thinner struts and a lower strut-vessel ratio. This could explain Biomatrix’s preferable distributions for all stresses analyzed in this study and agrees with similar research^10^ (Fig. 6).

The regions of highest WSS were located at the strut tips, which align to the flow, with low WSS recorded adjacent to the struts, which is also consistent with previous findings.^1^ Sites immediately downstream of strut intersections have also been identified as sites of abnormal flow.^14^

### Clinical Implications

#### Strut Spacing

Strut spacing should be considered in conjunction with strut size when assessing hemodynamic performance. Larger spacing has a beneficial effect on flow and the adverse effect of larger strut size appears to be reduced in combination with larger strut spacing’s. This could have important implications for future stent design as strut sizes introduces important mechanical considerations such as flexibility, vessel conformability, deployment recoil.^34^

#### Strut Size

In a similar manner, reducing strut size appears to have beneficial hemodynamic effects. Here, the TAWSS threshold analysis with a cut-off near the mode of the stress distribution was potentially misleading, and this may have contributed to the variability previously reported in the literature.^25^ Consideration of the stress distribution may provide better insight when considering physiological and pathophysiological responses.^21^

#### Stent Protrusion

Thrombogenetic risk associated with stent under-sizing is well recognized^43^ and adverse high WSS regions, which are associated with atherosclerotic plaque destabilization,^6^ were identified for the fully malapposed stent. Over-sizing stents may increase adverse compressive forces on the vessel^19^ but it has been demonstrated for the first time that the degree of protrusion may have adverse hemodynamic effects. Further, hemodynamic profiles introduced by stent design are then secondarily influenced by clinical deployment. Ideally, stents should be fully embedded in the vessel wall to avoid adverse hemodynamic effects on one hand, but this must be well balanced against high vessel wall tension on the other.^19^ Precise deployment is technically difficult with current clinical techniques, but imaging tools such as optical coherence tomography are able to detect stent apposition and may have an important future role in deployment.^35^

#### Stent Design Comparison

Mechanically desirable design features often have undesirable hemodynamic effects, requiring a balanced optimization. For example, increasing the number of peaks provides more scaffolding but also lowers TAWSS adversely. Similarly, thicker struts create a stronger stent, yet adversely affect WSS and WSSG. From the Omega and Biomatrix comparison, it was demonstrated how mechanically beneficial design attributes can be implemented with their undesirable hemodynamic effects being mitigated using other hemodynamically desirable design features.

### Study Limitations

Our study had several limitations. First, the idealized geometries were rigid, straight and atherosclerotic plaque was not included. However, idealized models have been found to be adequate for the investigation of straight geometries without plaque^31^ and lesion specific curvature or plaque deposition may prohibit observations exclusively of stent design. Further, a rigid- wall assumption has been demonstrated to be a reasonable simplification.^4^ Secondly, neglecting the local vessel deformation results in overestimation of the hemodynamic parameters, depending on the stent design.^27^ This variation may be amplified *in*-*vivo* where lesion type, stent design and deployment parameters result in a unique patient specific scenario. The present results provide an indication of the hemodynamic performance of stent design features only. Thirdly, cross-sectional shape was set to circular for all experiments, and comparison with other cross-sections is an area for future study. Beneficial effects of circular cross-sections have been demonstrated.^16^ Finally, it should be noted that stenting is a complex biological process depending on the vessel characteristics and disease stage, stent type, deployment technique, and pathophysiological tissue responses which are still not fully understood—a complex interdependent system which cannot be fully captured by entirely by CFD.

## Conclusion

This study describes the effect of major stent design considerations including strut spacing, stent size and luminal protrusion (with full malapposition) on hemodynamic stress and extends this analysis to compare two commercially available stent designs. As stents must be deployed in a wide range of vessels, these simplified geometries provide data with generic applicability in a range of clinical situations. Within the stated assumptions, we have shown how the adverse hemodynamic effect of specific design features (for example strut size) can be mitigated when combined with other hemodynamically favorable design features. We also demonstrated that stent protrusion rates worsened the stent’s hemodynamic profile, especially when fully malapposed. Thus, this study delivers useful data and guidelines on interacting hemodynamic effects of stent design and demonstrates the importance of stent apposition when considering stent introduced coronary hemodynamics. This may contribute in part to understanding stent thrombosis and restenosis following PCI with stent implant.
